# Dissecting the dynamics of virus-derived DNA of dengue virus 2 (DENV-2) in *Aedes* mosquitoes

**DOI:** 10.1371/journal.pone.0332245

**Published:** 2025-09-12

**Authors:** Hiroka Aonuma, Aboubacar Sombié, Jian-Chiuan Li, Manabu Ote, Erisha Saiki, Hidetoshi Ichimura, Itoe Iizuka, Taichi Odagawa, Tatsuya Sakurai, Kayoko Yamaji, Masayuki Saijo, Chun-Hong Chen, Athanase Badolo, Hirotaka Kanuka

**Affiliations:** 1 Department of Tropical Medicine, The Jikei University School of Medicine, Tokyo, Japan; 2 Center for Medical Entomology, The Jikei University School of Medicine, Tokyo, Japan; 3 Laboratoire d’Entomologie Fondamentale et Appliquée, Université Joseph Ki-Zerbo, Ouagadougou, Burkina Faso; 4 National Institute of Infectious Diseases and Vaccinology, National Health Research Institutes, Zhunan, Taiwan; 5 Laboratory Animal Facilities, The Jikei University School of Medicine, Tokyo, Japan; 6 Department of Virology 1, National Institute of Infectious Diseases, Tokyo, Japan; Guangzhou University, CHINA

## Abstract

Dengue is one of the neglected tropical diseases (NTDs) transmitted by *Aedes* mosquitoes and continues to spread globally. When mosquitoes are infected with dengue virus (DENV), virus-derived DNA (vDNA) is generated in mosquitoes, which subsequently contributes to their immune response. We traced the generation and presence of dengue virus type 2 (DENV-2) vDNA in experimentally infected cultured mosquito cells and *Aedes* mosquitoes, and notably, in wild mosquitoes collected in Burkina Faso. Detection of vDNA was achieved using a method incorporating loop-mediated isothermal amplification (LAMP), specifically, a LAMP-based vDNA detection method (vDNA-LAMP). The LAMP reaction, using primers targeting a segment of the NS5 region of DENV-2, detected vDNA from crude DNA extracted from experimentally infected cultured cells and *Aedes* mosquitoes. Detection revealed that the amount of DENV-2 vDNA generated in infected cells was relatively low; nevertheless, vDNA-LAMP enabled successful detection. The timing and quantity of vDNA generation in cultured cells were associated with the initial number of viral particles introduced during infection. Furthermore, vDNA-LAMP was applied to detect dengue virus vDNA in wild mosquitoes in dengue-endemic regions. This resulted in the successful detection of DENV-2 vDNA in field-collected mosquitoes, indicating that a proportion of wild mosquitoes in Burkina Faso harbored DENV-2 vDNA. Mapping these vDNA-positive mosquitoes allowed the identification of areas where infected mosquitoes and/or their progeny were likely present. These findings provide insights into the dynamics of DENV-2 vDNA in natural environments and underscore the potential of vDNA-LAMP as a tool for tracing vDNA in wild mosquitoes, which are responsible for transmitting viral infections.

## Introduction

Arthropod-borne viruses (arboviruses) including dengue, yellow fever, chikungunya, and Zika viruses, pose public health threats in tropical and subtropical locations [1–4]. Dengue is a mosquito-borne disease caused by dengue virus (DENV) and is primarily transmitted by *Aedes aegypti* and, to a lesser extent, by *Aedes albopictus* [5]. There are four DENV serotypes (DENV-1, DENV-2, DENV-3, and DENV-4), each exhibiting distinct epidemiological patterns [6]. Dengue is endemic in over 100 countries, and multiple DENV serotypes often co-circulate within the same regions [1]. A study on the prevalence of dengue estimated that 3.9 billion people are at risk of infection [7]. In 2023, the number of dengue cases reached an all-time high, affecting more than 80 countries [8]. Persistent transmission, coupled with an unexpected surge in cases, led to a historic peak of more than 6.5 million cases and more than 7,300 dengue-related deaths [1].

When insects are infected with RNA viruses, virus-derived DNA (vDNA) is generated within their bodies [9–12]. DNA form of RNA viruses have been shown to be generated by endogenous reverse transcriptase activity in insect cells [9,12]. vDNA enhances the RNAi-mediated antiviral immune response by regulating the number of viruses within the mosquito, thereby enabling the mosquito to survive while retaining the virus [12]. Recently, vertical transfer of vDNA from experimentally infected female *Aedes* mosquitoes to their offspring was demonstrated [13]. The production of vDNA in experimentally infected mosquitoes suggests that vDNA is present also among naturally infected wild mosquitoes and their offspring, either as a trace of viral infection or a result of vertical transfer in wild populations.

To investigate the presence of vDNA in wild mosquito, a sensitive and reliable detection method is essential. Such a method allow for monitoring of vDNA in field-collected mosquitoes, providing insight into natural infections in mosquito populations. Various diagnostic approaches has been employed to detect DENV, including nucleic acid amplification techniques, Immunoassays, and CRISPR-based assays [14]. Loop-mediated isothermal amplification (LAMP) is a molecular diagnostic method that offers rapid results, isothermal reaction conditions, and high sensitivity [15]. LAMP uses 4–6 primers, and the reaction proceeds at constant temperature using *Bst* polymerase [16]. Due to the characteristics of *Bst* polymerase, LAMP exhibits higher sensitivity, comparable to or even surpassing that of qPCR [15,17]. LAMP has been widely applied to detect pathogens with DNA genomes such as parasites and bacteria [18,19]. In addition, reverse-transcription (RT)-LAMP, which incorporates transcriptase into the reaction, has been increasingly used for detecting RNA pathogens such as viruses [17,18,20]. The genomic DNA of parasites (filaria and malaria parasites) in mosquitoes has been detected using LAMP [21,22]. These findings suggest that LAMP is a feasible method for detecting and tracing vDNA in virus-infected mosquitoes. Moreover, since the amount of vDNA is expected to be lower than that of viral RNA [10], LAMP is anticipated to facilitate gaining insight into the dynamics of vDNA. Indeed, LAMP has been successfully used to detect Zika virus vDNA in infected *Aedes* mosquitoes [23].

This study provides a comprehensive examination of vDNA generation in cultured cells and mosquitoes experimentally infected with DENV-2. Notably, detection of vDNA in wild mosquitoes underscores its potential as a molecular target for vector surveillance.

## Materials and methods

### Ethics statement

The mouse blood used in this study was purchased directly from BioLASCO Taiwan Co., Ltd. (http://www.biolasco.com.tw). The company acts in accordance with the Guide for the Care and Use of Laboratory Animals, the regulations on live vertebral animal experiments, and the ARRIVE guidelines. Mosquito collection in households was approved by the National Ethical Research Committee of the Ministry of Health, Burkina Faso (No. 2017-9-146 of 12/09/2017). Signed informed consent was obtained from all householders included in the study before starting the field collection.

### Cell culture and virus infection

Dengue virus type 2 (DENV-2), D2/Hu/OPD030NIID/2005 (GenBank: LC111438), was used in this study. Other dengue serotypes used were as follows: dengue virus type 1 (DENV-1) (GenBank: AB178040.1), dengue virus type 3 (DENV-3) CH53489 (GenBank: DQ863638.1), and dengue virus type 4 (DENV-4) isolate TVP/360 (GenBank: KU513442.1). DENVs were passaged in Vero cells (ATCC) to generate virus stocks. *Ae. albopictus* C6/36 cells were infected with DENVs to obtain total DNA from infected cells. Vero cells were cultured in D-MEM (high glucose) (Fujifilm Wako Pure Chemical) supplemented with 10% fetal bovine serum (FBS), and penicillin-streptomycin. C6/36 cells were cultured in E-MEM (Sigma) supplemented with 10% FBS, MEM Non-Essential Amino Acids Solution (Thermo Fisher Scientific Inc.), and penicillin-streptomycin. C6/36 cells were seeded in culture flasks and incubated at 28°C before infection with DENVs. For vDNA detection, C6/36 cells were infected at a multiplicity of infection (MOI) of 10 or that indicated in the Results section. The infected cells were incubated at 28°C.

### Mosquito rearing and virus infection

A natural vector species that can transmit DENV, *Ae. aegypti* mosquito (the Higgs strain) was used in this study. Mosquito larvae were reared at 28°C. The larvae were fed on a mixture of yeast powder (Taiwan Sugar Corporation) and goose liver powder (#7573, NTN) in a 1:1 ratio or food for Koi fish (Hikari; Kyorin Co. Ltd., Japan). Adults were maintained on a diet of 10% sucrose solution in a constant temperature and humidity-controlled room (28°C and ~70% relative humidity) with a 12-hour light/dark cycle. DENV strains used for oral infection were as follows: DENV-1 Myanmar 3886201 strain (GenBank: AY726550.1), DENV-2 New Guinea-C strain (GenBank: M29095.1), DENV-3 98TW503 strain (GenBank: DQ675531.1), and DENV-4 H241 strain (GenBank: AY947539.1). A solution containing each virus strain (2 × 10^7^ PFU/ml) was mixed 1:1 with mouse blood and fed to female mosquitoes via a membrane-covered metal plate at 37°C for 30 minutes. Blood-engorged mosquitoes were maintained at 28°C for 10 days post-infection by feeding with sugar solution. These mosquitoes were killed, dried at room temperature for 12 hours, pooled by 10 mosquitoes in a tube, and stored at −20°C until DNA extraction.

### RNA extraction and cDNA synthesis

For the reverse transcription (RT)-LAMP experiment to determine the sensitivity and serotype specificity, viral RNAs were extracted from viral stocks using High Pure Viral Nucleic Acid Kit (Roche Ltd.), according to the manufacturer’s instructions, except for Proteinase K treatment. Extracted RNAs were used directly for RT-LAMP reactions. To prepare positive control DNA, cDNA was synthesized from DENV RNA as follows: 1 μg of extracted RNA dissolved in 8.5 μl of RNase-free water was incubated with a reaction mixture (3 μl of 40 μM Random Primers (Thermo Fisher Scientific Inc.), 6 μl of 5 × First-Strand Buffer (Thermo Fisher Scientific Inc.), 1.9 μl of 0.1 M DTT (Thermo Fisher Scientific Inc.), and 7.5 μl of dNTP Mixture (Takara Bio Inc.)) at 65°C for 5 min. Then, 0.6 μl of RNase inhibitor (Promega Co.) and 0.5 μl of M-MLV reverse transcriptase (Thermo Fisher Scientific Inc.) were added to the reaction mixture and incubated at 37°C for 90 minutes. For reverse transcription (RT)-qPCR and RT-LAMP to compare the sensitivity of each detection method (Table 3), viral RNA was isolated from cells using RNAiso Plus (#9108, TaKaRa) in accordance with the manufacturer’s instructions. cDNAs were synthesized from 4 μl of RNA solution using M-MLV Reverse Transcriptase (#28025013, Thermo Fisher Scientific Inc.) and 0.5 µM of a specific reverse primer (ACC ATT CCA TTT TCT GGC GTT) with a reaction volume of 10 μl. Synthesized cDNA was used for the qPCR reaction.

**Table 3:** vDNA detection by LAMP in C6/36 cells at each day post-infection of DENV-2.

	MOI of DENV-2	
dpi	1 × 10^−4^	1 × 10^−2^
1	– – –	– – –
2	– – –	– – –
3	– – –	– – –
4	– – –	+ – –
5	– – –	+ – –
6	– – –	+ – –
7	+ – –	+ – –

The number of symbols [+ and –]: the number of samples examined. + : detected, –: not detected. dpi: days post-infection. MOI: multiplicity of infection.

### DNA extraction

DNA was extracted from DENV-infected cultured cells and mosquitoes as follows. Cells or mosquitoes were homogenized with a plastic homogenizer in 100 μl of Buffer A (0.1 M Tris (pH 9.0), 0.1 M EDTA, 1% SDS, and 0.5% DEPC) and incubated at 70°C for 30 minutes. Subsequently, the homogenate was mixed with 22.4 μl of 5 M KOAc and incubated for 30 minutes on ice. The supernatant was collected by centrifugation at 20,400 × g for 15 min at 4°C and mixed with 45 μl of isopropanol. Precipitated DNA was collected after centrifugation at 20,400 × g for 20 min at 4°C, rinsed with 70% ethanol, and dried. Each DNA pellet was dissolved and diluted with TE to adjust the concentration so that 1 μl of solution contained DNA from 1 × 10^4^ (Fig 3B) or 3.2 × 10^3^ (Table 2, Table 3) infected cultured cells counted at the seeding, one five-hundredth of each virus-infected mosquito pool (Fig 3C), or one-fiftieth of each wild mosquito pool (Fig 4 and Table 4). One microliter of each DNA solution was used as a template for the LAMP reaction.

**Table 2:** Sensitivity of LAMP and qPCR in detecting DENV-2 RNA and vDNA.

	RNA		vDNA	
MOI of DENV-2	RT-qPCR	RT-LAMP	qPCR	LAMP
1 × 10^−1^	+ + +	+ + +	+ + –	+ + (+)
1 × 10^−2^	+ + +	+ + +	+ – –	+ + (+)
1 × 10^−3^	+ + +	+ + +	+ + –	+ + (+)
1 × 10^−4^	+ + +	+ + +	– – –	+ + +
1 × 10^−5^	+ + –	+ + –	– – –	– – –
1 × 10^−6^	– – –	(+) – –	– – –	(+) – –
1 × 10^−7^	– – –	(+) – –	– – –	– – –
1 × 10^−8^	– – –	(+) – –	– – –	– – –

The number of symbols [+, (+), and –]: the number of samples examined. + : detected in all three repeated reactions per one sample, (+): detected only once or twice in three repeated reactions per one sample, –: not detected in three repeated reactions per one sample. MOI: multiplicity of infection.

**Table 4:** Infection rates by location of mosquito collection.

Location of collection	Houses	Mosquitoes	Pools	Pool size	Positive pools	Infection rate* (95%CI)
1200 logements	70	228	112	1-13	6	26.52 (11.06–53.82)
Tabtenga	61	139	95	1-10	5	36.18 (13.70–77.61)
Goundry	13	30	19	1-5	1	33.24 (1.94–150.42)
Total	144	397	226		12	

* Infection rate was shown as per 1,000 mosquitoes.

CI: Confidence Interval.

**Fig 1:**
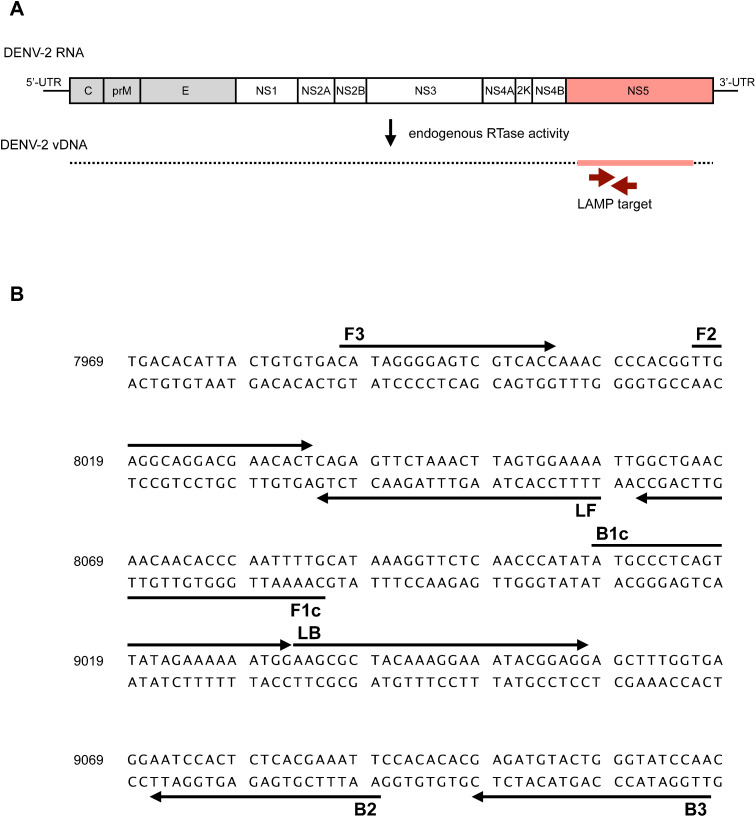
Schematic diagram of the DENV-2 RNA genome with LAMP primer positions for amplifying vDNA. (A) DENV-2 vDNA is generated from genomic RNA using endogenous RTase activity in mosquito cells. A set of arrows (brown) indicates the region of vDNA used as the target for the LAMP reaction. (B) Partial sequence of DENV-2 (GenBank: LC111438) with primer binding sites.

**Fig 2:**
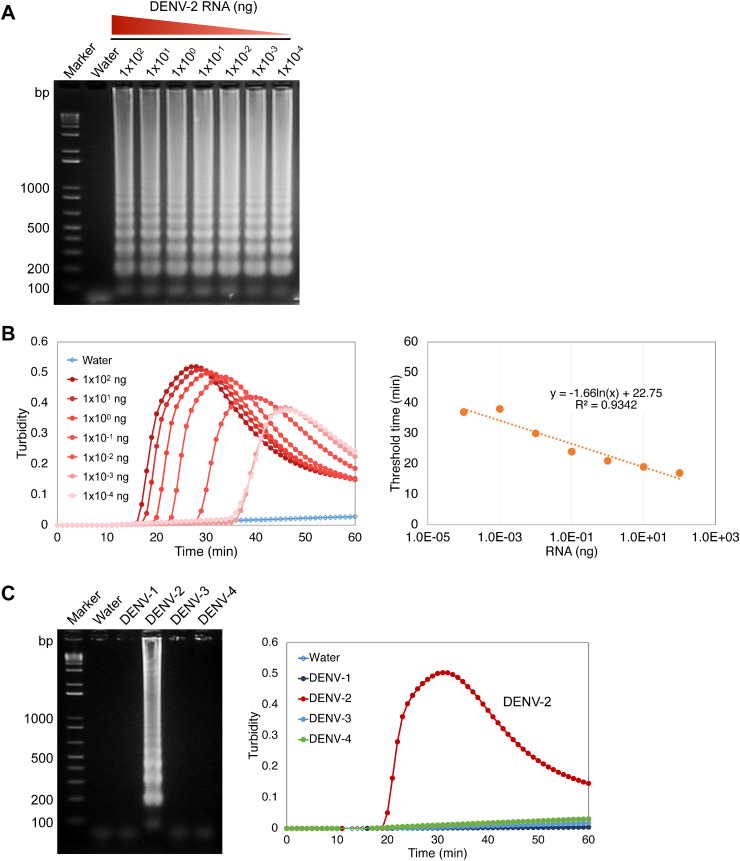
Sensitivity and serotype specificity of RT-LAMP for detecting DENV-2 genomic RNA. RNA of DENV-2 (1 × 10^2^, 1 × 10^1^, 1 × 10^0^, 1 × 10^−1^, 1 × 10^−2^, 1 × 10^−3^, and 1 × 10^−4^ ng) and DENV 1, 3, and 4 were used as a template for RT-LAMP reactions for 60 min at 58°C. Water served as a negative control. (A) Agarose gel electrophoresis of the RT-LAMP amplified products. Numbers on the left indicate migration of the molecular weight marker (bp). (B) (left) Amplification of DENV-2 RNA by RT-LAMP reaction monitored using a real-time turbidimeter. (right) Fitted curve based on quantities of RNA and the threshold time. The threshold time was determined as when turbidity reached greater than 0.05 by RT-LAMP reaction. (C) (left) Agarose gel electrophoresis of RT-LAMP amplified products with genomic RNA of DENV-1, 2, 3, and 4, showing the specificity of the reaction. The reaction mixtures were electrophoresed in a 2% agarose gel. Numbers on the left indicate migration of the molecular weight marker (bp). (right) Specific amplification of DENV-2 RNA monitored using a real-time turbidimeter.

**Fig 3:**
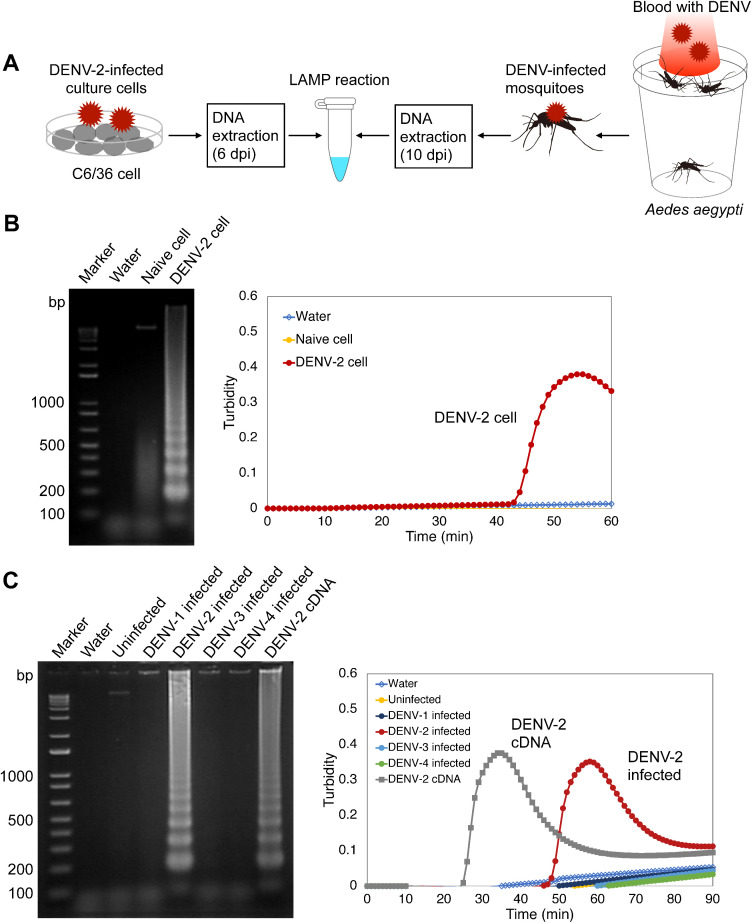
Detection of DENV-2 vDNA produced in mosquito cultured cells. (A) Scheme for preparing DENV-infected culture cells and mosquitoes for vDNA detection. Ten infected mosquitoes were pooled in each group and subjected to DNA extraction. One-fiftieth of the extracted DNA per group was used as the template for the LAMP reaction. dpi, days post- infection. (B) DENV-2 vDNA production in infected cells and detection using the LAMP reaction. (left) The reaction mixtures were electrophoresed on a 2% agarose gel. Numbers on the left indicate migration of the molecular weight marker (bp). (right) Amplification of vDNA in infected cells monitored using a real-time turbidimeter. (C) vDNA of DENV-2 in infected mosquitoes was specifically detected using LAMP. (left) Amplified products were electrophoresed in 2% agarose gels. DENV-2 cDNA was used as the positive control. Water served as a negative control. Numbers on the left indicate migration of molecular weight marker (bp). (right) Amplification of vDNA in infected mosquitoes monitored using a real-time turbidimeter. DENV-2 cDNA was used as a positive control.

**Fig 4:**
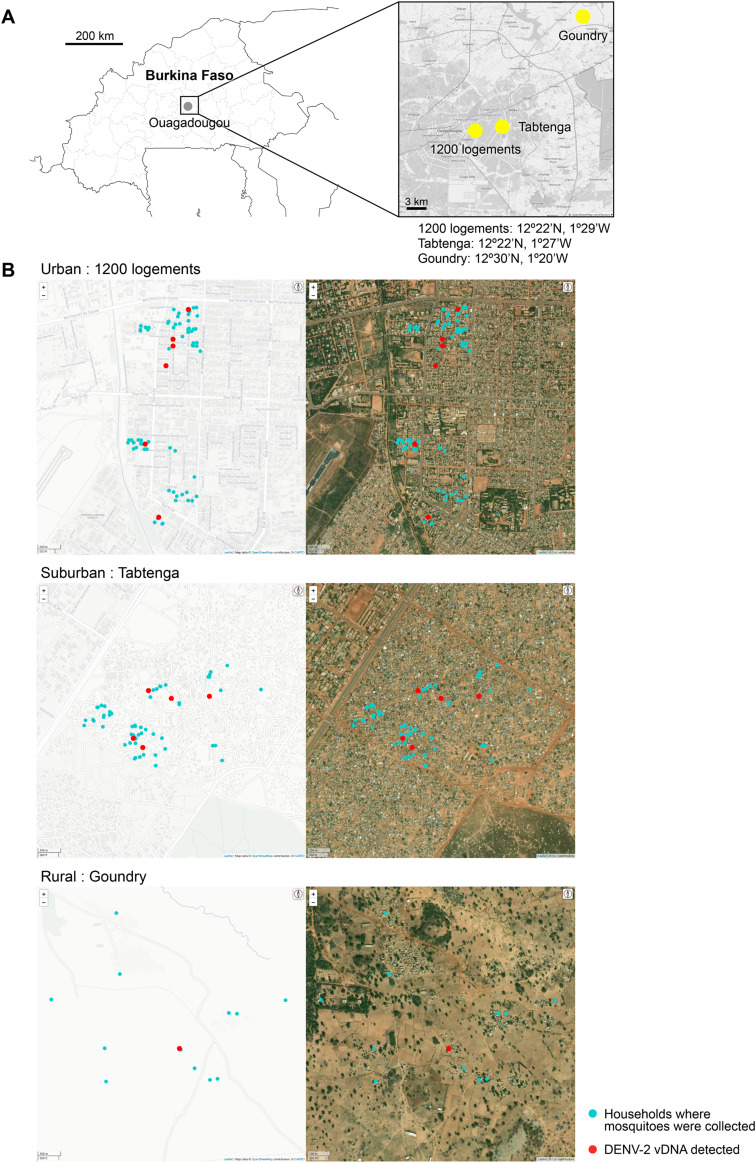
DENV-2 vDNA detection in wild mosquitoes collected in endemic locations. (A) Location of mosquito collection sites showing the capital city Ouagadougou (gray circled) and the study localities (colored squares) in Burkina Faso. (B) Spatial distribution of the households where mosquitoes were collected in three localities. Blue dots indicate households where mosquitoes were collected. Red dots indicate where DENV-2 vDNA was detected from the mosquitoes. Street map (left), aerial map (right). Plots on maps were created using LeafletR and OpenStreetMap (openstreetmap.org). Map data © OpenStreetMap contributors. Imagery © Esri, Maxar, Earthstar Geographics, and the GIS User Community.

### LAMP primer design

The LAMP primer set was designed based on information from 40 variations of DENV-2 full genome sequences (25 from Taiwan and 15 from African countries). All these sequences (GenBank: AJ968413.1, AY776328.1, DQ645540.1, DQ645541.1, DQ645542.1, DQ645543.1, DQ645544.1, DQ645545.1, DQ645546.1, DQ645547.1, DQ645548.1, DQ645549.1, DQ645550.1, DQ645551.1, DQ645552.1, DQ645553.1, DQ645554.1, DQ645555.1, DQ645556.1, KU365901.1, KU365902.1, KU365903.1, KJ734727.1, HQ891023.1, HQ891024.1, GU131843.1, KY627763.1, KY627762.1, EU056810.1, EF105389.1, EF105390.1, EF105382.1, EF105386.1, EF105385.1, EF457904.1, EF105384.1, EF105387.1, EU003591.1, EF105388.1, EF105378.1) were aligned as cDNA, and regions with high homology among those sequences were selected. Candidate sequences appropriate for primer design were determined using PrimerExplorer V5 (Fujitsu Ltd.). Several primer sets were designed and subject to RT-LAMP detecting RNA of a DENV-2 strain (D2/Hu/OPD030NIID/2005, GenBank: LC111438.1). One of the primer sets that amplified a smaller amount of template in a shorter time was selected and used in this study (Fig 1 and Table 1). The reaction temperature for the primer set was optimized to be 58°C and used in the subsequent experiments.

**Table 1:** Sequences of LAMP primers targeting DENV-2 NS5 region. Primers were designed based on the genome sequences of 40 variations of DENV-2.

Primer	Sequence (5’ → 3’)
F3	CATAGGGGAGTCGTCACC
B3	TTGGATACCCAGTACATCTC
FIP (F1c-F2)	CAAAATTGGATGTTGTTGTTCAACCTAGAAGCAGGACGAACACT
BIP (B1c-B2)	ATGCCCTCAGTCATAGAAAAAATGGAGTTTCGTGAGAGTGGATT
LF	TTTTCCACTAAGTTGAGGACTCTG
LB	AAGCACTACAAAGGAAATATGGAGG

### LAMP reaction

LAMP and RT-LAMP reactions were performed using a DNA Amplification Kit and an RNA Amplification Kit, respectively, in accordance with the manufacturer’s instructions (Eiken Chemical Co., Ltd.), except that half the default volume was used for the reaction. Each reaction was performed in a total volume of 12.5 μl reaction mixture containing 20 pmol of each FIP and BIP primer, 2.5 pmol of each F3 and B3 primer, 10 pmol of each Loop F and Loop B primer, and 6.25 μl 2 × reaction mixture (Table 1). For DNA amplification, 1 μl extracted DNA solution and 0.5 μl *Bst* DNA polymerase were added to the reaction mixture. For the RT-LAMP experiment to determine the sensitivity and serotype specificity, 2.5 μl extracted RNA solution and 0.5 μl enzyme mix (*Bst* DNA polymerase and AMV reverse transcriptase) were added to the reaction mixture. For RT-LAMP to compare the sensitivity of each detection method, 4 µl of RNA solution was used. Each reaction mixture was incubated at 58°C for 60 min or 90 min and terminated by incubation at 80°C for 5 min using a Loopamp Realtime Turbidimeter (LoopampEXIA, Eiken Chemical Co., Ltd.). Amplified reaction products were examined by electrophoresis in 2% agarose gels. The gels were stained with ethidium bromide and visualized under UV light.

### Quantitative PCR

The qPCR reactions were performed with 2 μl of the cDNA obtained or DNA extracted from infected cells, which was combined with TaqMan Fast Advanced Master Mix (#4444557, Thermo Fisher Scientific Inc.), 900 nM of the forward primer (GAR AGA CCA GAG ATC CTG CTG TCT), and 250 nM of the MGB probe (FAM-AGC ATC ATT CCA GGC AC) in a reaction volume of 20 μl [24]. The qPCR reactions were performed using a StepOnePlus PCR system (Thermo Fisher Scientific Inc.) with the following cycling conditions: 2 min at 50°C and 20 s at 95°C, followed by 40 cycles of 1 s at 95°C and 20 s at 60°C. The probe and all primers were purchased from a commercial company (Integrated DNA Technologies Inc.). The same primers and probe were used for the qPCR analysis of DNA samples.

### Mosquito collection

Wild mosquitoes were collected in or near Ouagadougou, the capital of Burkina Faso, an endemic location for DENV-2. Mosquito collections were conducted between August 15 and November 1, 2017. Three collection sites were established in 3 villages, 1200 logements (12  °22’N, 1 °29’W), Tabtenga (12  °22’N, 1 °27’W), and Goundry (12  °30’N, 1 °20’W), selected for differences in ecological characteristics, human population size, and housing type. Consent forms were obtained from households in each village. Mosquitoes were collected inside and outside the houses of approximately 1,000 households by aspiration. The collected mosquitoes were killed and transported to the laboratory of Université Joseph Ki-Zerbo, Ouagadougou, Burkina Faso, in a dry, ambient-temperature environment. Mosquitoes were identified to the species level using a microscope at room temperature, and each mosquito pool was assigned an ID number. The mosquitoes were then stored at −30°C. The locations of households where mosquitoes were collected were recorded based on Global Positioning System (GPS) data. Households where mosquitoes were collected and the households from which positive mosquitoes were obtained were plotted on maps using Leaflet 1.7.1 [25] and OpenStreetMap data (openstreetmap.org). The base map was generated using OpenStreetMap data (© OpenStreetMap contributors) and Esri World Imagery (© Esri, Maxar, Earthstar Geographics, and the GIS User Community). Contains information from OpenStreetMap and OpenStreetMap Foundation, which is made available under the Open Database License (https://www.openstreetmap.org/copyright).

### Statistical analysis

The infection rate (IR) of mosquitoes was estimated using PooledInfRate version 4.0 (CDC) [26]. The number of individuals and positive/negative vDNA-LAMP results for each pool were entered into the worksheet. The output included the bias-corrected maximum likelihood estimate (MLE) and 95% skewness-corrected score confidence interval. The IR was expressed as the number of infections per 1,000 individuals.

## Results

### RT-LAMP-based validation of primers detecting DENV-2 cDNA

In this study, DENV-2, responsible for an outbreak in Burkina Faso in 2017, was selected as the target of vDNA-LAMP among the four DENV serotypes. In evaluating primer sets to detect DENV-2 vDNA, we employed the conventional RT-LAMP method since RT-LAMP involves making cDNA using reverse transcriptase, and cDNA can be considered a surrogate for vDNA. At first, the genome sequences of forty DENV-2 strains reported from dengue-endemic countries were aligned, and several genomic regions of high homology were focused on designing LAMP primer sets. Multiple primer sets targeting different regions of the DENV-2 genome were designed and screened. Among these, the primer set targeting the NS5 region yielded the most favorable results in terms of sensitivity and amplification efficiency across a range of reaction temperatures and RNA concentrations (Fig 1 and Table 1). Based on these findings, the reaction conditions for this primer set were subsequently optimized.

To evaluate the primer set that was later used for DENV-2 vDNA detection via LAMP, serially diluted DENV-2 RNA (1 × 10^2^, 1 × 10^1^, 1 × 10^0^, 1 × 10^−1^, 1 × 10^−2^, 1 × 10^−3^, and 1 × 10^−4^ ng) was used as a template. RNAs extracted from the supernatant of infected cultured cells were subjected to the RT-LAMP. In optimized conditions (58°C for 60 min), viral cDNA transcribed from RNA was detected down to 1 × 10^−4^ ng RNA (equivalent to approximately 1.74 × 10^4^ copies of DENV-2 genome RNA) within 40 min of incubation (Fig 2A). Similar results were also confirmed by measuring white turbidity caused by magnesium pyrophosphate, a by-product of amplification (Fig 2B). The threshold time of amplification was determined as when turbidity reached greater than 0.05 by RT-LAMP reaction. A fitted curve based on the quantities of RNA and the threshold time was obtained (x: RNA quantity (ng), y: threshold time (min), y = −1.66ln(x)+22.75, R^2^ = 0.9342), which allows for the estimation of viral RNA quantity from the threshold time obtained in the LAMP reaction (Fig 2B).

The specificity of the primer set detecting DENV-2 was then evaluated by the ability to distinguish between the RNAs of the other three DENVs. Viral RNAs extracted from DENV types 1–4 were used to test the specificity of the LAMP reaction. Amplification reactions were performed using equal amounts of each RNA as the template by RT-LAMP with the DENV-2-specific primer set. Only DENV-2 was amplified, but not DENV-1, 3, or 4, as determined by real-time turbidimetry and electrophoresis (Fig 2C). These demonstrated that a primer set suitable for detection of the DENV-2 cDNA has been obtained, which is applicable for vDNA-LAMP assay detecting DENV-2 vDNA.

### LAMP detection of vDNA in DENV-2-infected cells and mosquitoes

To examine whether DENV-2 vDNA can be detected by LAMP reaction using the validated primer set, a mosquito cell line was used. C6/36 cells (*Aedes albopictus* clone) were infected with DENV-2 at MOI 10 followed by extracting DNA, not RNA, from the infected cells 6 days post-infection (Fig 3A). Each DNA sample was used as a template to detect intracellular DENV-2 vDNA by LAMP reaction. As a result, DENV-2 vDNA was detected specifically from infected cells (Fig 3B), consistent with previously reported results [6]. The observation that the elevation in turbidity occurred 45 minutes after the initiation of the reaction suggests a relatively limited production of DENV-2 vDNA in these infected cells (equivalent to approximately 2.63 × 10^2^ copies of DENV-2 genome RNA per reaction tube, based on the standard curve of viral RNA quantity and amplification time shown in Fig 2B).

Next, the production of vDNA in experimentally infected mosquitoes was examined using the vDNA-LAMP method. The serotype-specificity of DENV-2 vDNA-LAMP was also evaluated using four DENV serotypes. Female *Aedes aegypti* were orally infected with each DENV type 1–4 through artificial membrane feeding of infected blood. At 10 days post-infection, the mosquitoes were killed, dried, and subjected to DNA extraction and LAMP reaction (Fig 3A). As a result, only mosquitoes from the DENV-2 infected group were identified as positive by the DENV-2 vDNA-LAMP reaction (Fig 3C). The quantity of DENV-2 vDNA generated in the mosquito is estimated to be very low, corresponding to approximately 2.37 × 10^1^ copies of DENV-2 genome RNA/a reaction tube, similar to that observed in the cell (Figs 2B and 3C). These data demonstrated that DENV-2 vDNA is generated in infected mosquitoes and can be amplified by the LAMP reaction.

### Comparison of LAMP and qPCR for DENV-2 vDNA detection

The sensitivity of LAMP for detecting DENV-2 vDNA was evaluated and compared with that of quantitative PCR (qPCR), a commonly used viral detection method. C6/36 cells were infected with DENV-2 using a range of MOIs. On day 7 post-infection, cells were harvested for RNA and DNA extraction. Detection of DENV-2 RNA by RT-qPCR was robust up to infected samples at MOI 1 × 10^−4^; at MOI 1 × 10^−5^, RNA was detected in two samples and not in one, indicating a detection limit of MOI 1 × 10^−5^. When the same RNA samples were subjected to RT-LAMP, DENV-2 RNA was detected in all samples at MOI 1 × 10^−4^ and 2 of 3 at MOI 1 × 10^−5^ (Table 2), indicating that the sensitivity of RT-LAMP was comparable to that of RT-qPCR for detecting DENV-2 RNA. The detection limit of DENV-2 vDNA by LAMP was subsequently examined. vDNA amplified by qPCR exhibited a detection threshold of MOI 1 × 10^−3^, but LAMP, in contrast, demonstrated the amplification of DENV-2 vDNA in samples up to MOI 1 × 10^−4^ (Table 2). These results indicate that LAMP is comparable to or more sensitive than qPCR for DENV-2 vDNA detection. RNA tends to be more readily detectable than vDNA in the type of template applied to LAMP.

To determine the timing of DENV-2 vDNA synthesis in mosquito cells, C6/36 cells were infected with DENV-2 at either MOI 1 × 10^−4^ or MOI 1 × 10^−2^, the MOI at which infected cells can be harvested alive after a certain number of days (Fig 3A). DNA was extracted from the infected cells, which were collected at intervals ranging from 1 to 7 days post-infection (dpi) and subjected to vDNA-LAMP analysis. As a result, in cells infected at MOI 1 × 10^−4^, DENV-2 vDNA was detected at 7 dpi. On the other hand, in cells infected at MOI 1 × 10^−2^, vDNA was detected starting from 4 dpi (Table 3 and S1 Fig). These data suggested that vDNA becomes detectable a few days after infection, correlating with the escalation of intracellular virus above a certain level.

### LAMP detection of DENV-2 vDNA in wild mosquitoes

Finally, we investigated whether DENV-2 vDNA is present and detectable in wild mosquitoes collected in dengue-endemic areas. Burkina Faso, a landlocked country in West Africa, is one of the most dengue-endemic countries on the African continent, notably marked by a severe outbreak in the capital city of Ouagadougou in 2017, wherein over 10,000 cases were reported [27]. Within this region, the *Aedes aegypti* mosquito emerged as the primary vector for the dengue virus, exhibiting a preference for human hosts [28]. In this study, wild *Aedes* mosquitoes were collected inside and outside the houses of consenting households in three distinct locations (urban, suburban, and rural) in or near Ouagadougou (Fig 4A). In total, 397 mosquitoes were divided into 226 pools by household, with some pools corresponding to the same household. DNA, not RNA, was extracted from dead, desiccated mosquitoes and was subjected to DENV-2 vDNA-LAMP.

As a result, amplified vDNA sequences were detected in 12 pools, suggesting the presence of dengue virus-infected mosquitoes within the mosquito populations collected from 12 out of the 144 households. The locations of infected mosquitoes within each location were identified by plotting the residences where mosquitoes were collected. Those where infected mosquitoes were identified using GPS (Fig 4B). The vDNA-based DENV-2 mosquito infection rates (per 1,000 mosquitoes) were estimated for urban (1200 logements), suburban (Tabtenga), and rural (Goundry) locations, yielding rates of 26.52 (95%CI = 11.06–53.82), 36.18 (95%CI = 13.70–77.61), and 33.24 (95%CI = 1.94–150.42), respectively (Table 4). To the best of our knowledge, this marks the first evidence of arbovirus vDNA detection in wild mosquitoes. These findings indicated locations where mosquitoes infected with DENV-2 are prevalent, may be posing an elevated risk of transmission to humans.

## Discussion

In this study, DENV-2 vDNA was detected in cultured cells, experimentally infected mosquitoes, and, notably, in naturally infected wild mosquitoes, using LAMP, a highly sensitive technique for tracking vDNA generated in their bodies. The most significant finding is that, similar to laboratory-reared mosquitoes, wild mosquitoes also generate vDNA of an arbovirus after infection. These wild mosquitoes are suspected to have either fed on infected blood in their natural environment or acquired the virus and/or vDNA vertically from their parent mosquitoes.

vDNA was first discovered in *Drosophila* infected with the Flock House virus [9], and its generation has since been observed in other insect species. Information about from when vDNA is generated and can be detected after viral infection is crucial for understanding the dynamics of vDNA. In this study, mosquito cells were infected with DENV-2 at two different MOIs, followed by the subsequent identification of vDNA post-infection to elucidate the temporal dynamics of vDNA synthesis within mosquito cells. The results suggested an association between the vDNA detection in mosquito cells at specific time points and the initial number of viral particles introduced during infection. It is predicted that the timing of vDNA production in vector mosquitoes also depends on the viral load ingested through infected blood. However, notable variations in vDNA detection were observed among cells harvested on the same day post-infection (Table 2 and Table 3). These results suggest potential heterogeneity in the timing, quantity, and spatial distribution of vDNA generation, even among cells or mosquitoes infected in similar conditions. This is similar to the significant variability observed among individuals in the degree of antiviral protection of offspring of virus-infected *Drosophila* and *Aedes* mosquitoes [29]. Comparative analysis of viral RNA and vDNA revealed that the quantity of vDNA generated in cells is minute, approximately two orders of magnitude lower than that of viral RNA.

In this study, we performed both LAMP and RT-LAMP using a primer set targeting the NS5 region of DENV-2. The primer set was designed with the aim of detecting vDNA in mosquitoes, assuming field collection in both Taiwan and Africa. Using this primer set, RT-LAMP detected RNA corresponding to 1.74 × 10^4^ viral copies; RNA quantities below this threshold were not tested. In contrast, LAMP analysis of vDNA from experimentally infected mosquitoes detected a quantity equivalent to 2.37 × 10^1^ viral copies. This sensitivity is consistent with a previous report demonstrating the detection of DENV-2 RNA at 10^1^ viral copies by RT-LAMP [17]. Primer sets designed for broad detection of diverse variant sequences are often limited by the designable region, which may result in reduced sensitivity [30–32]. Primer design focused on specific variants may help further enhance sensitivity [32].

It is anticipated that specific regions within the viral genome serve as hotspots for producing vDNA, implying a targeted synthesis of vDNA in these areas. In this study, we employed a primer set designed for detecting the NS5 region based on its sensitivity. To comprehensively track vDNA production across various regions of the virus’s genome, additional analyses using primer sets specific to other regions are needed. Concurrently, employing qPCR and the LAMP technique to detect and compare vDNA production across distinct regions enables the identification and analysis of hotspots for vDNA production.

When using cultured cells, this study limited the follow-up of infected cells to day 7 due to the impact on cell viability. Considering the survival period during which mosquitoes engage in blood-feeding and their ability to transmit the virus, it is expected that viral replication continues within the mosquito body beyond the seventh day of infection, leading to increased vDNA production. Longitudinally tracking infected mosquitoes and detecting vDNA within their bodies would provide important information for estimating the temporal dynamics of vDNA production in wild mosquitoes.

Detection of DENV-2 vDNA in certain wild mosquitoes collected in Burkina Faso demonstrated the presence of vDNA in natural settings. Amplification was observed only in a limited number of mosquito pools collected in Burkina Faso, rather than broadly across all mosquitoes. This indicates that the viral sequence targeted by the primers is unlikely to have been integrated into the mosquito genome, but rather produced in a mosquito after they had infected with DENV-2, at least in the study area. Furthermore, the locations where the mosquitoes were collected facilitated the identification of sites where DENV-2 vDNA-positive mosquitoes were distributed. This information was used to determine the infection rate of mosquitoes at each location. Though the infection rate observed in our study was relatively low, DENV infection among wild mosquitoes in Burkina Faso has generally been reported to be low. This aligns with previous data obtained using the qRT-PCR method [33]. However, it is essential to note that, as previously discussed, the estimated vDNA amount in infected mosquitoes is considerably lower than that of viral RNA. Infection rates determined based on vDNA detection are likely to be underestimated, suggesting that the actual infection rate in mosquitoes is substantially higher. In other words, the positive detection of vDNA indicated the existence of more infected mosquitoes, implying a significantly higher actual infection rate.

Based on previous reports of mosquito surveys and the findings obtained in this study, it is evident that the direct detection of viral RNA stands out as the most sensitive and reliable method for virus detection in mosquitoes. Accordingly, viral RNA detection has been employed as the standard approach in mosquito virus surveillance [34,35]. On the other hand, it is important to emphasize that the wild mosquitoes collected and used in this study were preserved in a deceased and desiccated state. Consequently, vDNA-based detection represents a survey method that is not affected by the preservation conditions of collected mosquitoes. This feature proves advantageous for practical survey efforts in endemic locations where the handling of RNA is logistically challenging. Moreover, the vDNA-LAMP method facilitates the amplification of target vDNA in isothermal conditions, eliminating the need for costly equipment. This method is anticipated to streamline the process of mosquito monitoring in areas endemic to arbovirus-borne diseases.

This study has limitations that need to be addressed in future investigation. Whether mosquitoes harboring vDNA actually contain the virus in their saliva and can subsequently establish infections was not examined in this study. Therefore, the detection of vDNA alone does not imply that the mosquito is capable of transmitting infectious viruses. In other words, mosquitoes in which vDNA was detected in this study have ingested the virus and consequently generated vDNA, but this only indicates traces of infection or result of vertical transfer, rather than current infectivity or transmission capacity. It is important to note that vDNA serves as a marker of viral presence in the area rather than a definitive indicator of a mosquito’s current capacity to transmit the virus. Further study is needed to clarify the relationship between vDNA production in individual mosquitoes, the presence of infectious virus in their saliva, and their vector competence.

## Conclusions

In this study, we investigated the dynamics of dengue virus vDNA by analyzing both experimentally infected mosquitoes and wild populations from dengue-endemic regions. Our results confirmed the presence of vDNA in wild mosquitoes under natural conditions, suggesting its potential circulation in the environment. By mapping vDNA-positive mosquitoes, we identified key areas where infected mosquitoes and/or their progeny were likely to persist. These findings provide critical insights into the transmission dynamics of mosquito-borne viruses and offer valuable information to guide future vector control strategies.

## Supporting information

S1 Fig Agarose gel electrophoresis of LAMP products corresponding to Table 3. (PDF)

S1 Raw Images Original blot and gel images contained in the manuscript’s figures. (PDF)

S2 Data Excel file containing the raw data used to generate all figures and tables in the manuscript. (XLSX)
