# Supplementary material for: Dissecting the dynamics of virus-derived DNA of dengue virus 2 (DENV-2) in Aedes mosquitoes
Source: PLoS One. 2025 Sep 12;20(9):e0332245. doi: 10.1371/journal.pone.0332245 (PMC12431436; doi:10.1371/journal.pone.0332245)
Supplement: S1 Fig — (PDF) [file pone.0332245.s001.pdf]

S1 Fig

MOI 1×10^{-4}

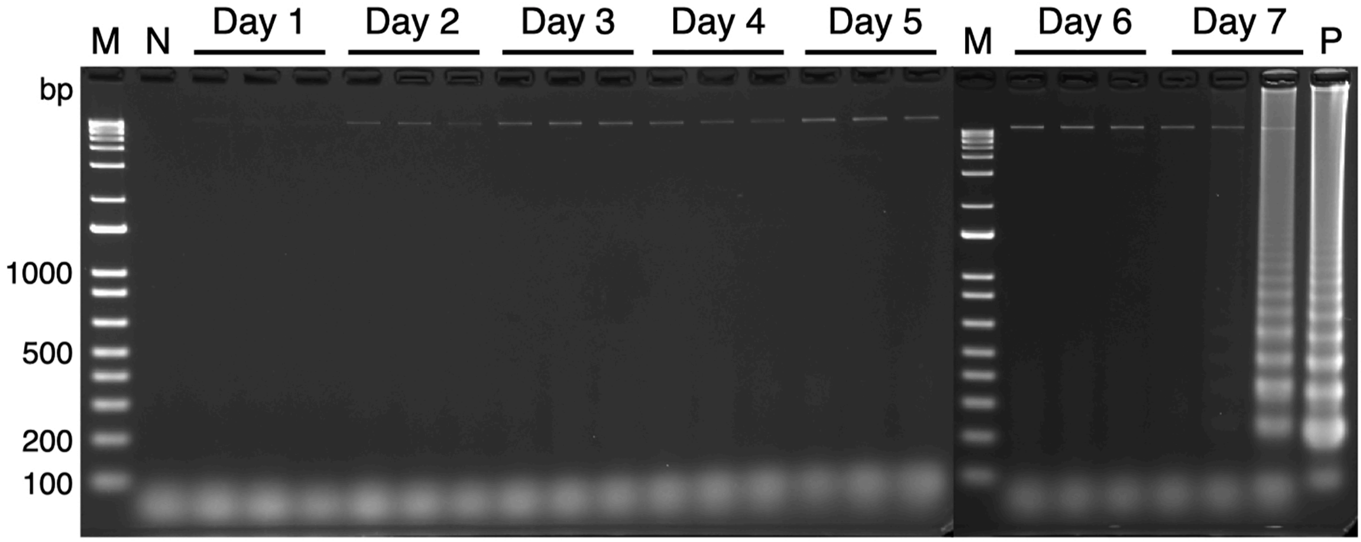

MOI 1×10^{-2}

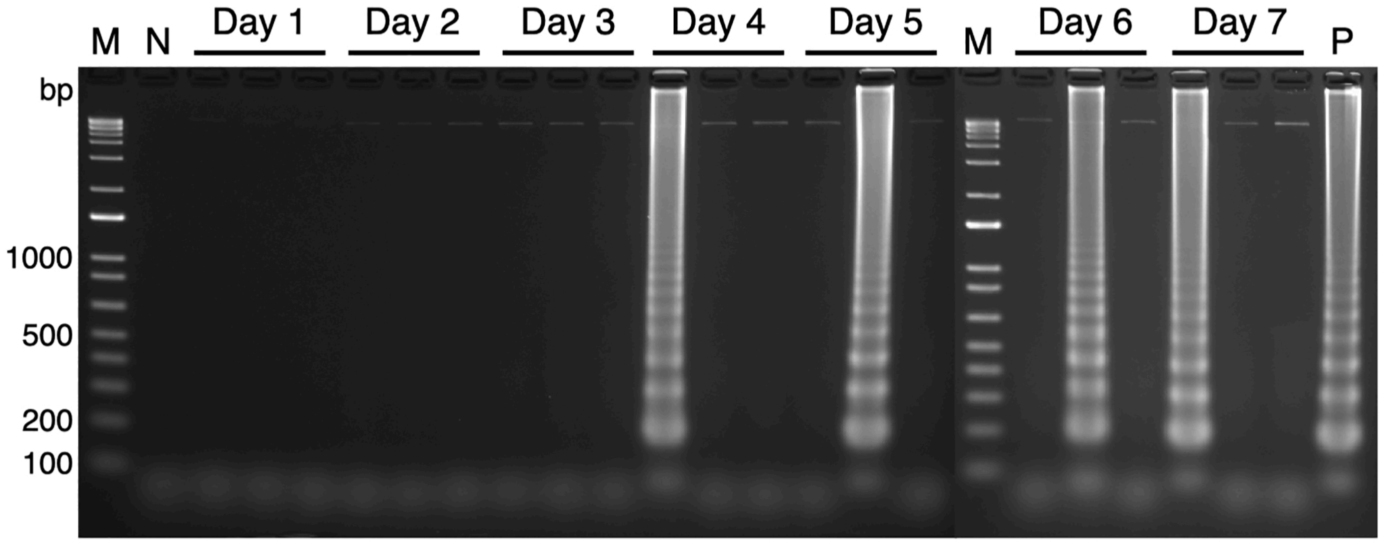

Agarose gel electrophoresis of LAMP products corresponding to Table 3. DENV-2 vDNA generated in infected cells at a multiplicity of infection (MOI) of 1×10^{-4} or 1×10^{-2} was detected by LAMP. M: DNA ladder; N: Water (negative control); P: DENV-2 cDNA (positive control).
