# Supplementary material for: Dissecting the dynamics of virus-derived DNA of dengue virus 2 (DENV-2) in Aedes mosquitoes
Source: PLoS One. 2025 Sep 12;20(9):e0332245. doi: 10.1371/journal.pone.0332245 (PMC12431436; doi:10.1371/journal.pone.0332245)
Supplement: S1 Raw Images — (PDF) [file pone.0332245.s002.pdf]

S1_raw_images

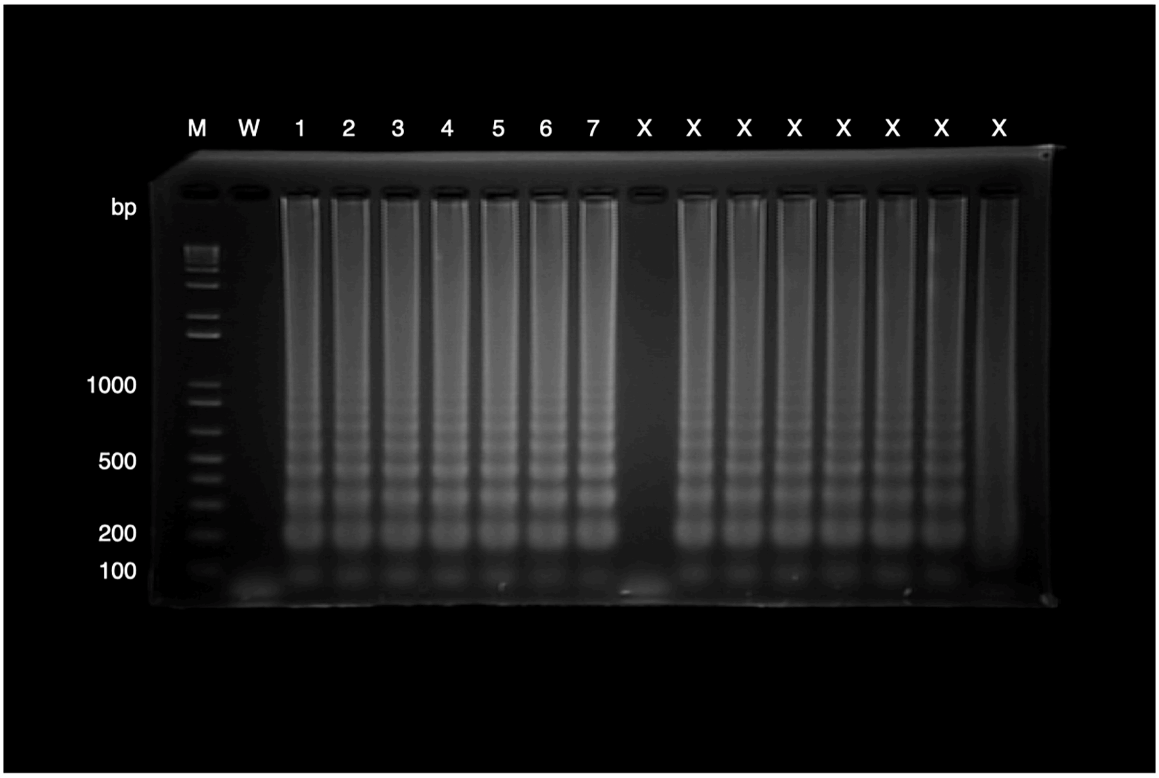

Raw image of the 2% agarose gel corresponding to Fig 2A. The primer set was evaluated by detecting DENV-2 genomic RNA. Lane M: DNA ladder; Lane W: water (negative control); Lanes 1–7: amplification products obtained using serially diluted DENV-2 RNA (1×10^{2}, 1×10^{1}, 1×10^{0}, 1×10^{-1}, 1×10^{-2}, 1×10^{-3}, and 1×10^{-4} ng, respectively).

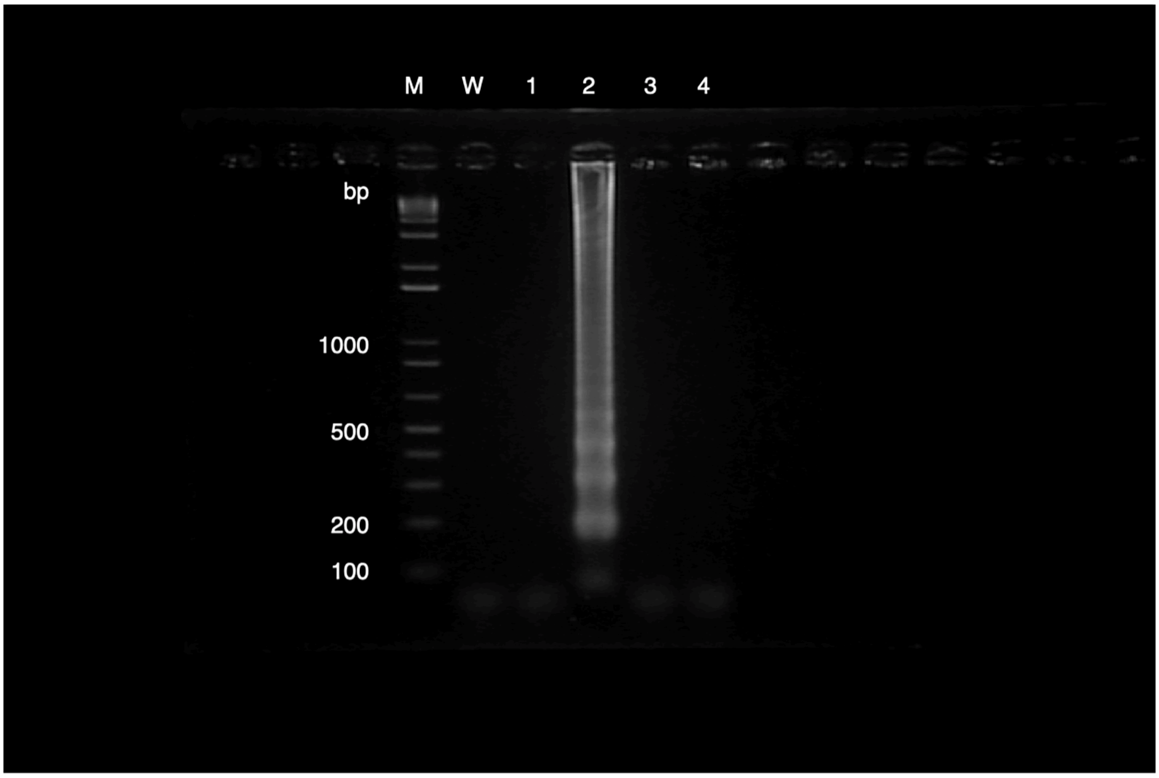

Raw image of the 2% agarose gel corresponding to Fig 2C. The specificity of the primer set for DENV-2 detection was evaluated. Lane M: DNA ladder; Lane W: water (negative control); Lanes 1–4: amplification results obtained using genomic RNA from DENV- 1, 2, 3, 4, respectively.

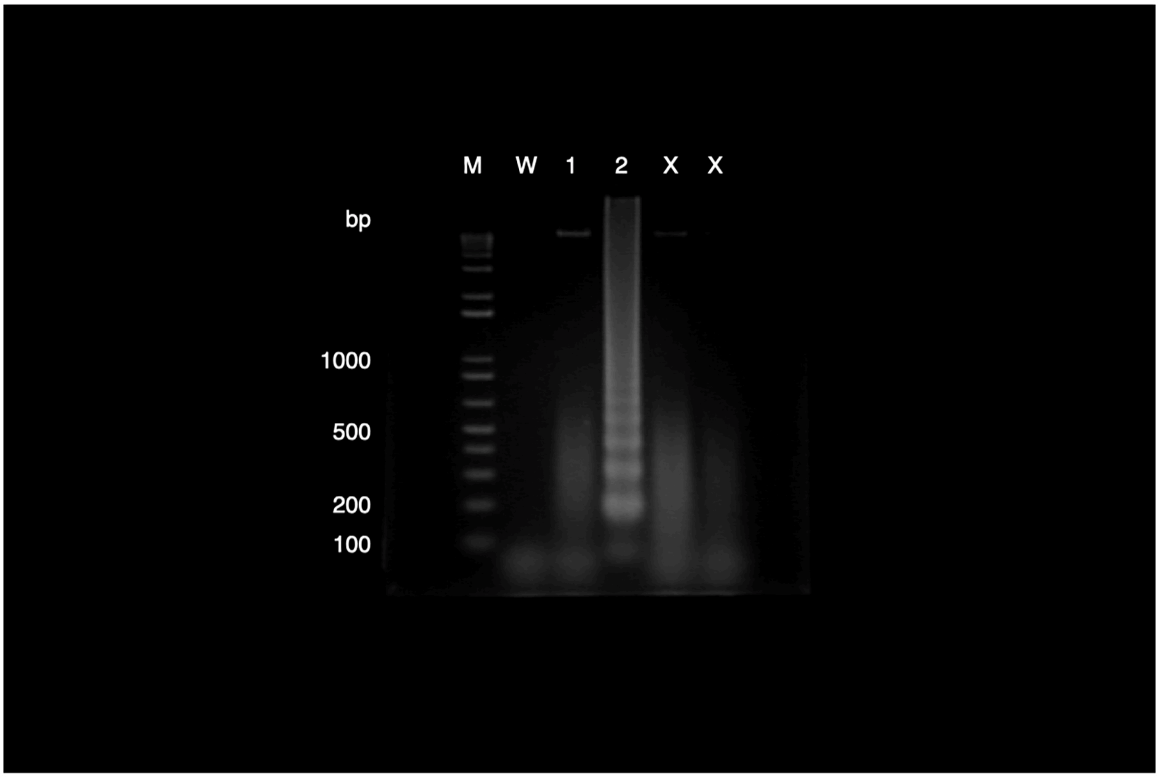

Raw image of the 2% agarose gel corresponding to Fig 3B. DENV-2 vDNA produced in infected cells was detected by LAMP. Lane M: DNA ladder; Lane W: water (negative control); Lanes 1–2: amplification results using DNA extracted from uninfected cells and DENV-2 infected cells, respectively.

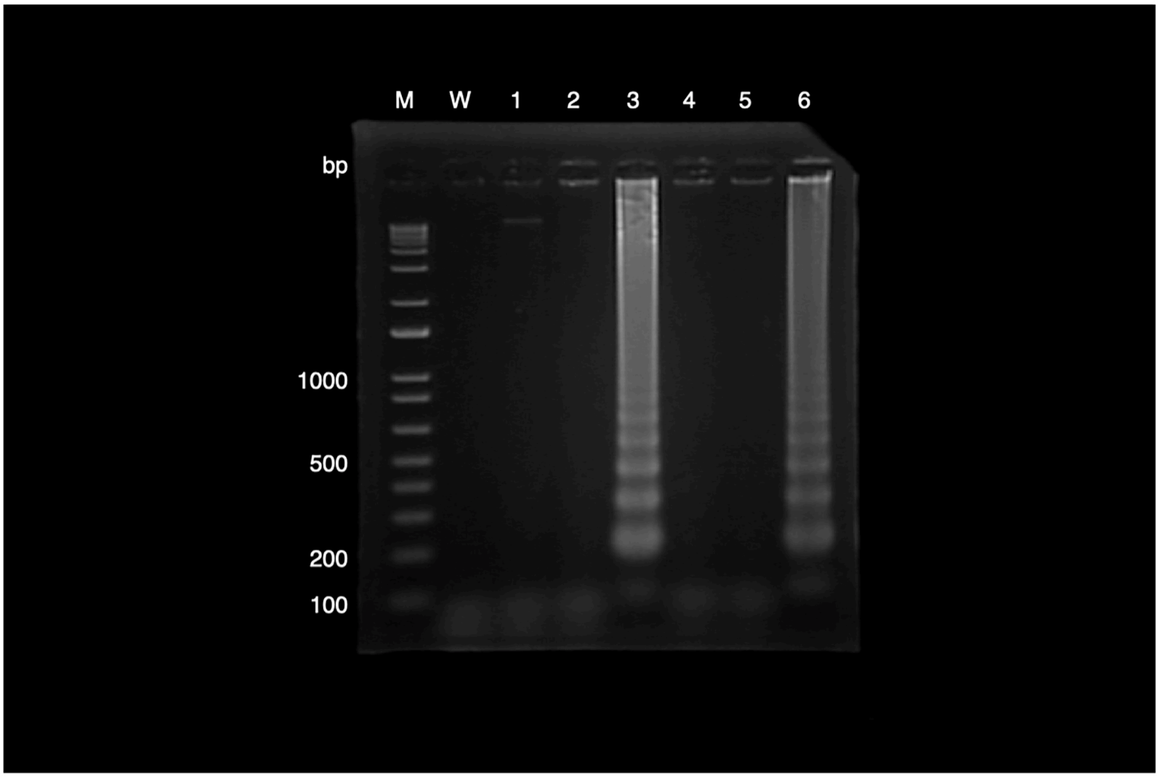

Raw image of the 2% agarose gel corresponding to Fig 3C. DENV-2 vDNA produced in infected mosquitoes was detected by LAMP. The specificity of the LAMP reaction was demonstrated by the selective amplification of DNA extracted from DENV-2-infected mosquitoes. Lane M: DNA ladder; Lane W: water (negative control); Lanes 1–5: amplification results using DNA extracted from uninfected, DENV-1-infected, DENV-2-infected, DENV-3-infected, DENV-4-infected mosquitoes, respectively. Lane 6: DENV-2 cDNA (positive control).

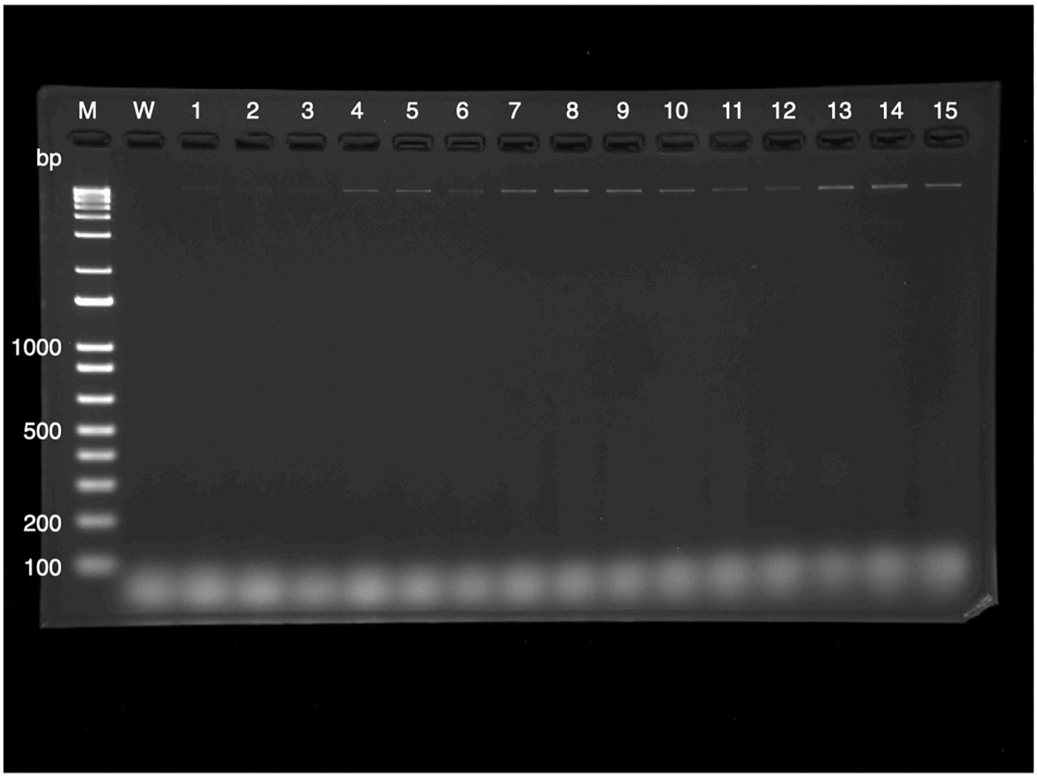

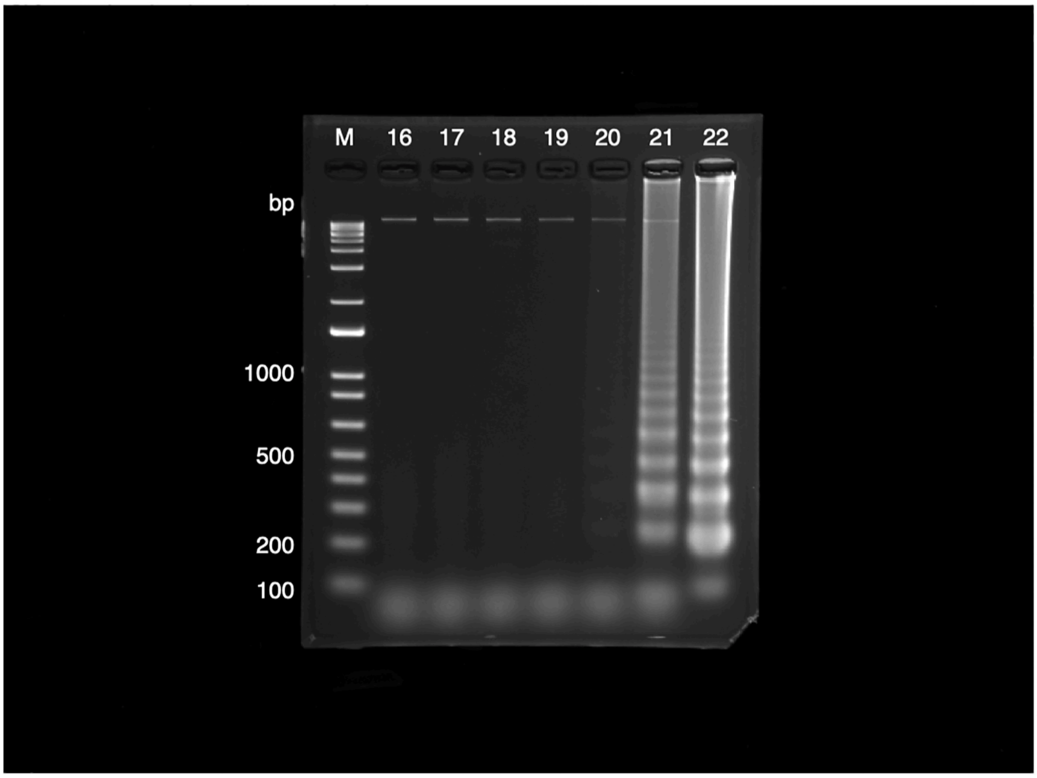

Raw images of the 2% agarose gel corresponding to S1 Fig. DENV-2 vDNA produced in infected cells (MOI = 1 × 10^{-4}) was detected by LAMP. Lane M: DNA ladder; Lane W: water (negative control); Lanes 1–21: amplification products collected over seven consecutive days post-infection. Each set of three lanes corresponds to samples from day 1 to day 7 (i.e., lanes 1–3: day 1, lanes 4–6: day 2, …, lanes 19–21: day 7). Lane 22: DENV-2 cDNA (positive control).

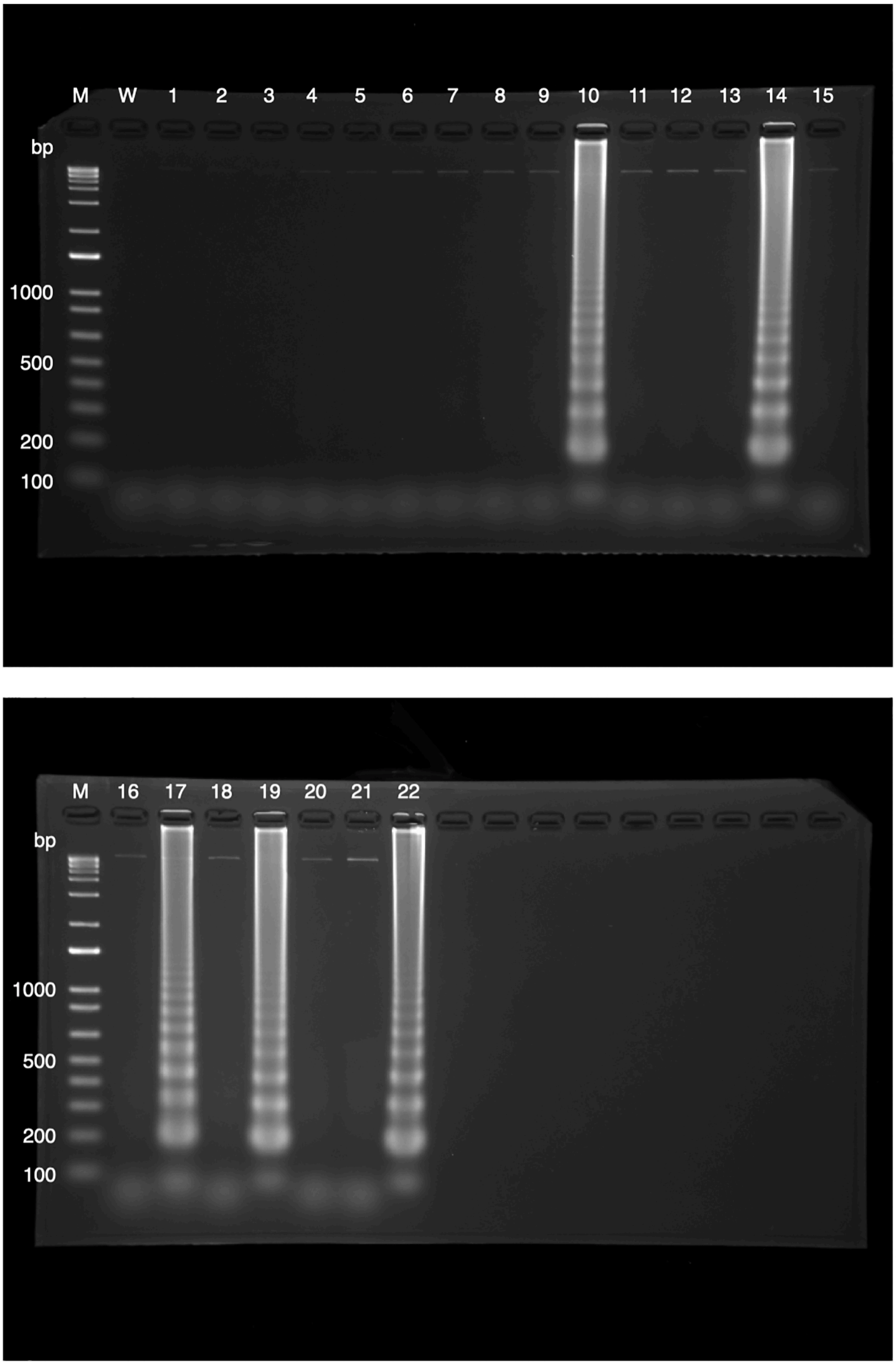

Raw images of the 2% agarose gel corresponding to S1 Fig. DENV-2 vDNA produced in infected cells (MOI = 1×10^{-2}) was detected by LAMP. Lane M: DNA ladder; Lane W: water (negative control); Lanes 1–21: amplification products collected over seven consecutive days post-infection. Each set of three lanes corresponds to samples from day 1 to day 7 (i.e., lanes 1–3: day 1, lanes 4–6: day 2, …, lanes 19–21: day 7). Lane 22: DENV-2 cDNA (positive control).
